# Correction: Wang et al. Identification and Verification of a Novel MAGI2-AS3/miRNA-374-5p/FOXO1 Network Associated with HBV-Related HCC. *Cells* 2022, *11*, 3466

**DOI:** 10.3390/cells14040268

**Published:** 2025-02-13

**Authors:** Chao Wang, Kunkai Su, Hanchao Lin, Beini Cen, Shusen Zheng, Xiao Xu

**Affiliations:** 1 Key Laboratory of Integrated Oncology and Intelligent Medicine of Zhejiang Province, Department of Hepatobiliary and Pancreatic Surgery, Affiliated Hangzhou First People’s Hospital, Zhejiang University School of Medicine, Hangzhou 310006, China; skqweasd1080@zju.edu.cn (C.W.); 22111220001@m.fudan.edu.cn (H.L.); 15156859199@163.com (B.C.); 2 Westlake Laboratory of Life Sciences and Biomedicine, Hangzhou 310024, China; 3 NHC Key Laboratory of Combined Multi-Organ Transplantation, Hangzhou 310003, China; 4 Institute of Organ Transplantation, Zhejiang University, Hangzhou 310003, China; 5 State Key Laboratory for Diagnosis and Treatment of Infectious Diseases, National Clinical Research Center for Infectious Diseases, Collaborative Innovation Center for Diagnosis and Treatment of Infectious Diseases, The First Affiliated Hospital, School of Medicine, Zhejiang University, Hangzhou 310003, China; ksu@zju.edu.cn; 6 Department of Hepatobiliary and Pancreatic Surgery, The First Affiliated Hospital, Zhejiang University School of Medicine, 79 Qingchun Road, Hangzhou 310003, China

## Error in Figure

In the original publication [1], there was a mistake in Figure 9 as it was published. In Figure 9E and 9K, the panels representing the overexpression of cell migration conditioned by Lenti-NC and SI-NC were the same. The corrected Figure 9 appears below. The authors state that the scientific conclusions are unaffected. This correction was approved by the Academic Editor. The original publication has also been updated.

## Figures and Tables

**Figure 9:**
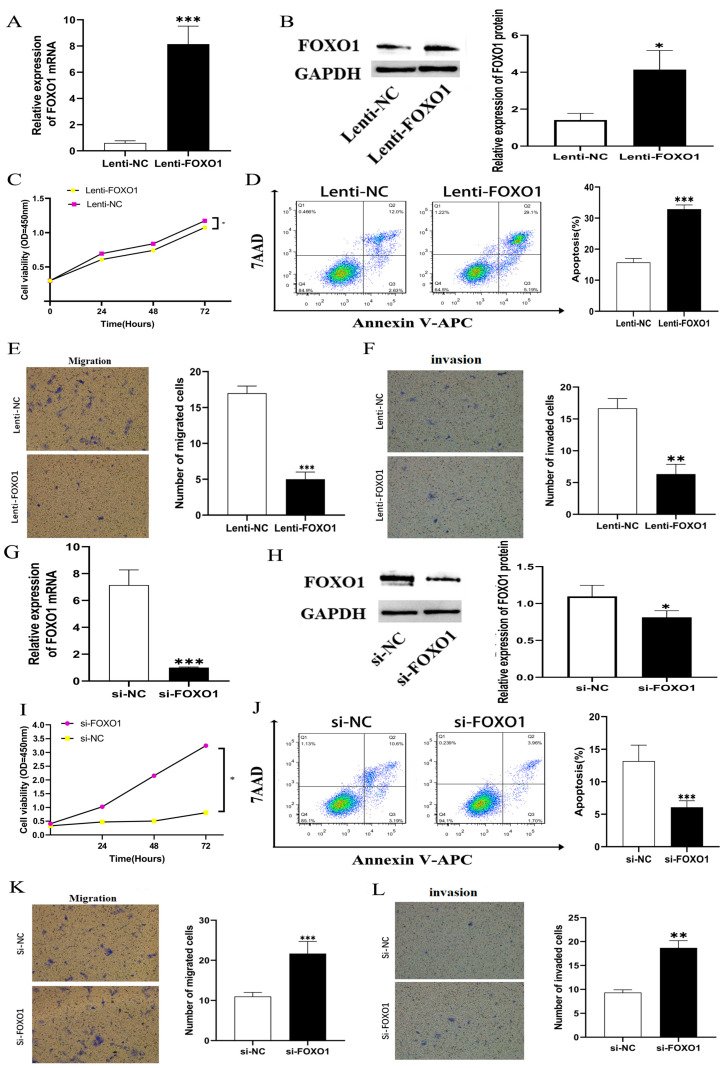
(**A**,**B**) QRT-PCR and western blot showed that expression of FOXO1 mRNA and protein were significantly upregulated in LM3 cells transfected with overexpressed vector. (**C**) Cell proliferation was decreased significantly in LM3 cells by overexpressing FOXO1; (**D**) A flow cytometry analysis indicated increased apoptosis induced by FOXO1 overexpression in LM3 cells. (**E**,**F**) A transwell test revealed overexpression of FOXO1 suppressed LM3 cell migration and invasion ability induced by MAGI2-AS3 overexpression in LM3 cells. (**G**,**H**) QRT-PCR and western blot showed that expression of FOXO1 mRNA and protein were significantly downregulated in LM3 cells with si-FOXO1. (**I**) LM3 cells with si-FOXO1 had increased viability of proliferation. (**J**) Flow cytometry analysis indicated decreased apoptosis of LM3 cells after knocking down FOXO1. (**K**,**L**) A Transwell test revealed that migration and invasion ability increased in LM3 cells by knocking down FOXO1. * *p* < 0.05, ** *p* < 0.01, *** *p* < 0.001. FOXO1, Forkhead Box O1. QRT-PCR: quantitative real-time polymerase chain reaction.
