# Supplementary figures and images for: Identification and Verification of a Novel MAGI2-AS3/miRNA-374-5p/FOXO1 Network Associated with HBV-Related HCC
Source: Cells. 2022 Nov 1;11(21):3466. doi: 10.3390/cells11213466 (PMC9654666; doi:10.3390/cells11213466)

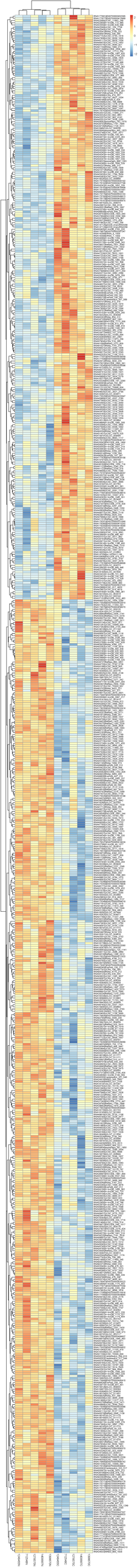

Supplement: Supplementary file 1 [file cells-11-03466-s001.zip › figure S1.tiff]

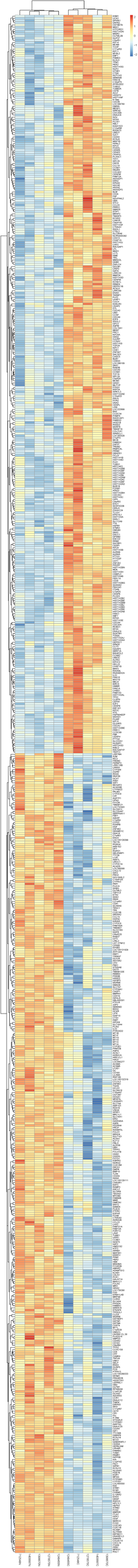

Supplement: Supplementary file 1 [file cells-11-03466-s001.zip › figure S2.tiff]

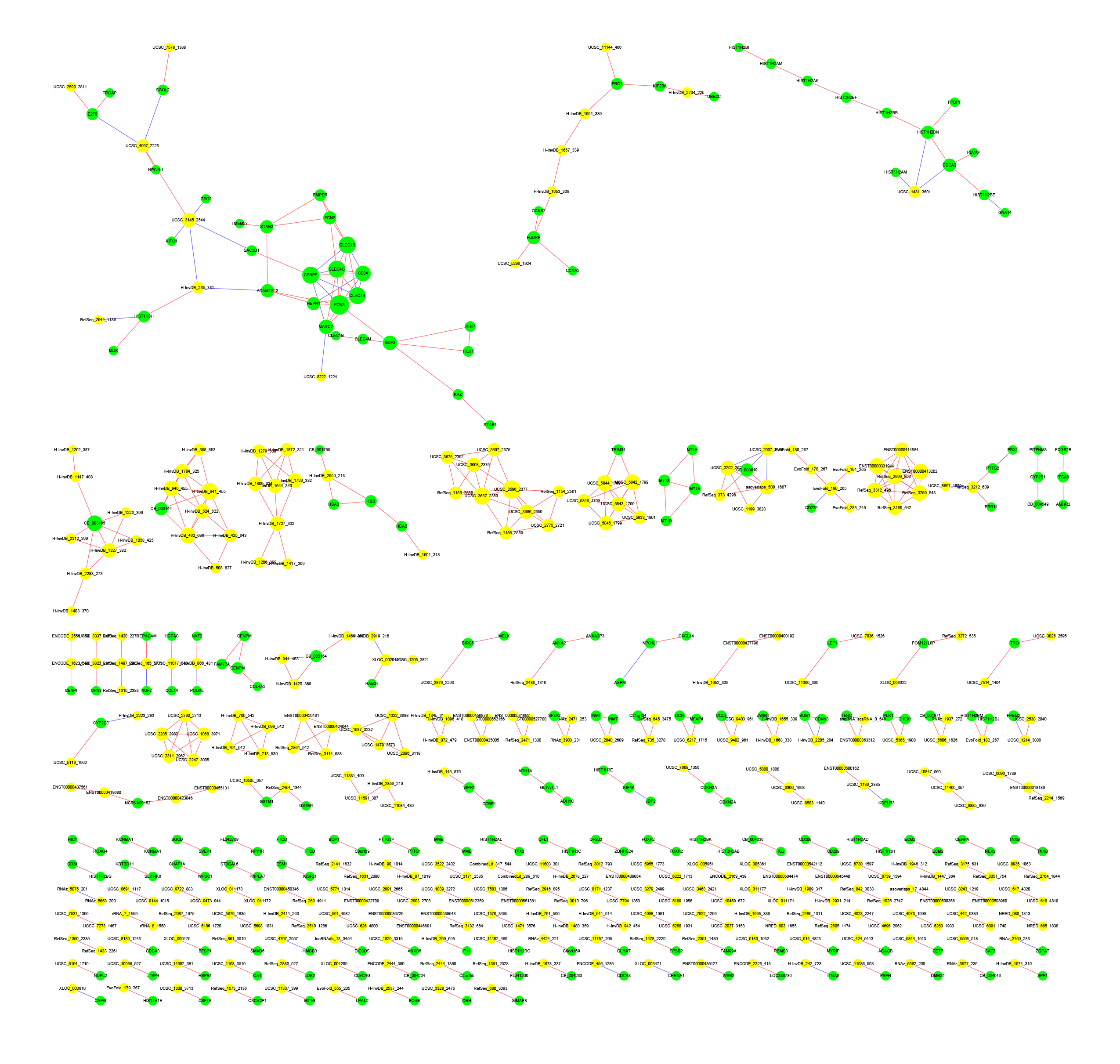

Supplement: Supplementary file 1 [file cells-11-03466-s001.zip › figure S3.tif]
